# CD9: A possible clue into breast cancer chemoresistance

**DOI:** 10.18632/oncotarget.27130

**Published:** 2019-08-13

**Authors:** Natalia Pacienza, Gustavo Yannarelli

**Affiliations:** Laboratorio de Regulación Génica y Células Madre, Instituto de Medicina Traslacional, Trasplante y Bioingeniería (IMeTTyB), Universidad Favaloro-CONICET, Buenos Aires, Argentina; Laboratorio de Regulación Génica y Células Madre, Instituto de Medicina Traslacional, Trasplante y Bioingeniería (IMeTTyB), Universidad Favaloro-CONICET, Buenos Aires, Argentina

**Keywords:** breast cancer, chemoresistance, mesenchymal stem cells, CD9

***News on: Mesenchymal stem cells confer chemoresistance in breast cancer via a CD9 dependent mechanism by Ullah et al. Oncotarget. 2019; 10:3435–3450. https://doi.org/10.18632/oncotarget.26952***

Breast cancer is the most common malignancy in women [1]. Resistance to chemotherapy accounts for the relapse that occurs in a proportion of patients [2]. Unfortunately, it is not possible to predict whether and when it will occur, and the mechanisms involved are still poorly understood. Chemoresistance can be an intrinsic characteristic of tumor cells or it can be acquired later on, despite an initial positive response to therapy. Acquired chemoresistance may arise from the interaction between cancer cells and the tumor microenvironment (TME), which contains mesenchymal stem/stromal cells (MSCs), endothelial cells, and tumor-associated macrophages and fibroblasts [2]. In fact, there is a growing amount of studies that supports the role of TME in the development of chemoresistance as well as in other processes related to cancer progression, such as the acquisition of an invasive phenotype, protection against anti-tumor immune response and neoangiogenesis. Thus, novel research in this field is currently focusing on the development of anti-cancer strategies targeted towards TME.

Among the 33 human tetraspanin proteins, CD9 plays particularly important roles in cancer. Although CD9 expression has been frequently observed in cancer tissue, its impact on tumor progression has recently received substantial attention. It has been shown that CD9 can modulate tumor cell growth, invasion, metastasis, and drug sensitivity [3–5]. In this context, the study by Ullah *et al*. [6] expands current knowledge by demonstrating a link between breast cancer cell chemoresistance and CD9 expression in MSCs. Their findings evidenced one of the possible interactions between stem cells and cancer cells that leads to the development of chemotherapy drug resistance. In this study, the administration of ERα-, PR-, and HER2-negative (triple negative) breast cancer cells (HCC1806 cell line) together with human bone marrow (BM)-derived MSCs into the mammary fat pad of NOD/SCID mice generated tumors containing a new hybrid cell. Interestingly, these hybrid cells, which have characteristics of both, cancer cells and MSCs, produced tumors that were reduced in size and exhibited increased resistance to the chemotherapeutic agents doxorubicin and 5-fluorouracil in comparison with tumors generated by HCC1806 cells [6]. The up-regulation of CD9 expression in hybrid cells was identified as the major difference against HCC1806 cells, along with the acquisition of multidrug resistance proteins. Noteworthy, experiments performed using CD9-silenced BM-MSCs generated hybrid cells that presented low levels of multidrug resistance proteins and thus, were sensitive to chemotherapy. The analysis of inflammatory factors in serum identified IL-6, CCL5, CCR5, and CXCL12 as possible mediators of the protective effects exerted by CD9 on cancer cells. These data indicate that chemoresistant tumor cells actively communicate with stem cells, as part of the tumor-associated microenvironment, through cytokines, chemokines, and CD9 [6]. CD9 is highly enriched in exosomes suggesting that its transference from MSCs to breast cancer cells may be mediated by this particular type of extracellular vesicles. Whether this interaction through CD9 occurs by a paracrine mechanism and/or requires direct cell-to-cell contact needs to be further investigated.

The study by Ullah *et al*. [6] infers that CD9 modulates the tumor response to chemotherapy and suggests that one potential method to overcome breast cancer chemoresistance is to design therapies with CD9-specificity. In this context, only a few reports demonstrated the benefits of blocking CD9 activity in chemotherapy-treated cancers [7, 8]. It has been shown that the inhibition of CD9 can activate T cells for triggering immune responses against tumor cells [5, 7]. In addition, the work by Ullah *et al.* [6] now directly links CD9 expression with tumor acquired chemoresistance. Interestingly, stem cells and cancer cells communicate via CD9. CD9 expression limits the tumor response to chemotherapy by up-regulating drug resistance proteins; notably, this effect requires the production of CXCL12. These observations add weight to the current notion that CD9 plays intertwined roles in chemoresistance.

By deciphering CD9 cues, further studies will explore how to break the cycle of chemoresistance and recurrence. Elucidating the underlying mechanisms of CD9 expression is still needed to better design effective CD9-specific therapies to overcome chemoresistance. Initial results indicate anti-tumoral activity against breast cancer in mouse models. This type of targeted therapy is feasible to do. Recently, CD19-directed chimeric antigen receptor (CAR) T cell therapy has been approved by the FDA and added to the list of therapies for B cell malignancies [9]. The clinical testing of CD9-targeting agents, however, has yet to deliver on its promise.
